# Experience of nutritional counselling in a nutritional programme in HIV care in the Tigray region of Ethiopia using the socio-ecological model

**DOI:** 10.1186/s41043-021-00256-9

**Published:** 2021-07-28

**Authors:** Fisaha Haile Tesfay, Anna Ziersch, Lillian Mwanri, Sara Javanparast

**Affiliations:** 1 Institute for Health Transformation, Deakin University, Geelong, Melbourne, Australia; 2 Southgate Institute for Health, Society and Equity, Flinders University, Adelaide, Australia; 3 School of Public Health, Mekelle University, Mekelle, Ethiopia; 4 College of Medicine and Public Health, Flinders University, Adelaide, Australia

**Keywords:** Nutritional counselling, Nutritional programme, Undernutrition, HIV, Tigray, Ethiopia

## Abstract

**Background:**

In many resource-poor settings, nutritional counselling is one of the key components of nutrition support programmes aiming to improve nutritional and health outcomes amongst people living with HIV. Counselling methods, contents and recommendations that are culturally appropriate, locally tailored and economically affordable are essential to ensure desired health and nutritional outcomes are achieved. However, there is little evidence showing the effectiveness of counselling in nutritional programmes in HIV care, and the extent to which counselling policies and guidelines are translated into practice and utilised by people with HIV suffering from undernutrition. This study aimed to explore these gaps in the Tigray region of Ethiopia.

**Methods and participants:**

A qualitative study was conducted in Tigray region Ethiopia between May and August 2016. Forty-eight individual interviews were conducted with 20 undernourished adults living with HIV and 15 caregivers of children living with HIV enrolled in a nutritional programme in three hospitals, as well as 11 health providers, and 2 programme managers. Data analysis was undertaken using the Framework approach and guided by the socio-ecological model. Qualitative data analysis software (QSR NVivo 11) was used to assist data analysis. The study findings are presented using the consolidated criteria for the reporting of qualitative research (COREQ).

**Result:**

The study highlighted that nutritional counselling as a key element of the nutritional programme in HIV care varied in scope, content, and length. Whilst the findings clearly demonstrated the acceptability of the nutritional counselling for participants, a range of challenges hindered the application of counselling recommendations in participants’ everyday lives. Identified challenges included the lack of comprehensiveness of the counselling in terms of providing advice about the nutritional support and dietary practice, participants’ poor understanding of multiple issues related to nutrition counselling and the nutrition programme, lack of consistency in the content, duration and mode of delivery of nutritional counselling, inadequate refresher training for providers and the absence of socioeconomic considerations in nutritional programme planning and implementation. Evidence from this study suggests that counselling in nutritional programmes in HIV care was not adequately structured and lacked a holistic and comprehensive approach.

**Conclusion:**

Nutritional counselling provided to people living with HIV lacks comprehensiveness, consistency and varies in scope, content and duration. To achieve programme goal of improved nutritional status, counselling guidelines and practices should be structured in a way that takes a holistic view of patient’s life and considers cultural and socioeconomic situations. Additionally, capacity development of nutritional counsellors and health providers is highly recommended to ensure counselling provides assistance to improve the nutritional well-being of people living with HIV.

**Supplementary Information:**

The online version contains supplementary material available at 10.1186/s41043-021-00256-9.

## Introduction

There is strong evidence on the interaction between undernutrition and HIV infection, with demonstrated negative impacts at individual and community levels. HIV infection and AIDS increase people’s predisposition to undernutrition [1]. Due to clinical, social, and economic factors, people living with HIV are more likely to suffer from undernutrition and its consequences [1–3]. Estimates of the prevalence of undernutrition amongst people living with HIV in sub-Saharan Africa varies—for example, from 19.4% in Tanzania [4] to 42.5% in Ethiopia [5]. International health agencies including the World Health Organisation (WHO) recommend nutritional care as an important component of HIV care services, particularly in resource-poor settings [6, 7].

Evidence suggests that a combination of antiretroviral therapy (ART) and nutritional care can improve adherence to ART and nutritional well-being. Ivers and colleagues found that adults living with HIV had better adherence to ART at 6 and 12 months of using nutritional supplementation respectively than those who were not provided with food assistance [8]. In addition, there is strong evidence on the benefits of nutritional support in terms of improving the nutritional status of people living with HIV [9–11].

To address HIV-related undernutrition and to improve HIV care outcomes, nutritional programmes have been incorporated into HIV care services in many countries, particularly in sub-Saharan Africa [11–14]. Nutritional programmes mainly comprise three components: (1) nutritional assessment; (2) nutritional support and (3) nutritional counselling. Nutritional assessment can involve a range of parameters but most commonly includes nutritional screening which measures key anthropometric indicators to guide the provision of different therapeutic and supplementary foods [11, 12, 15]. Food-based supplements such as high-energy ready-to-use therapeutic foods (RUTF) and corn-soy-blended (CSB) are the most commonly used nutritional supports in HIV care settings to treat protein-energy undernutrition [16]. The third component of nutritional programmes is nutritional counselling, which aims to assist patients to (a) maintain weight through increased energy intake; (b) practise safe infant feeding; (c) practise safe food or water handling and (d) manage HIV-related illness [15, 17].

Nutritional counselling plays a critical role in improving individuals’ nutritional knowledge and understanding of the importance of nutrition in their lives [18]. Such engagement through individual or group counselling sessions can empower patients to make informed decisions regarding their eating practice based on their financial circumstances and food availability and to take control over their nutritional circumstances [18, 19]. Culturally appropriate nutritional counselling involves tailored content to the local cultural context, incorporating local knowledge, expertise and lifestyle choices, distributing locally and culturally appropriate food and adapting the nutritional counselling to the local people’s educational level [19, 20].

The evidence regarding the availability and the practice of nutritional counselling in HIV care is inconsistent. Whilst a study in sub-Saharan African countries found the inclusion of nutritional counselling in 95% of the study sites [21], another study from Ghana reported a lack of nutritional counselling in HIV care programmes [22].

Evidence on the role of nutritional counselling in improving the nutritional status of people living with HIV is diverse [23–25]. Some studies have supported the importance of nutrition counselling, [24] but others are inconclusive about the benefits of counselling for the management of nutrition in HIV care. For example, a study conducted in Nigeria found that people living with HIV who were offered regular nutritional assessment and counselling had better nutritional outcomes than those who were not [25]. In contrast, a study from Brazil showed that the provision of nutritional counselling to undernourished people living with HIV and tuberculosis did not improve their nutritional status [23], though it is possible that tuberculosis infection might have undermined the positive impact of nutritional counselling. In a study from Uganda, nutritional counselling was found to be effective in improving the overall quality of life of people living with HIV, when given as a package of nutritional and other interventions such as nutritional support [26].

There is little evidence on the best techniques of counselling such as individual vs groups, frequency and duration of counselling and the possible roles of counsellors such as peers, nurses, physicians and other auxiliary health workers in HIV care settings [27].

### The nutritional programme in HIV care in Ethiopia

The nutritional programme in HIV care settings in Ethiopia commenced in 2010 and involves three main components to address the nutritional challenges of people living with HIV [12]. The programme involves nutritional assessment for all people living with HIV on ART and pre-ART, classification of nutritional status and enrolment of those undernourished into a nutritional programme. The nutritional support programme provides therapeutic food (Plumpynut) for 6 months for severely acute undernourished (SAM) and supplementary food (Plumpysup) for 3 months in moderately or mildly undernourished people living with HIV. Nutritional counselling is also one of the key components, similar to other areas in sub-Saharan Africa [11].

According to the national nutritional programme guidelines in HIV care in Ethiopia, nutritional counselling involves the provision of general information to people living with HIV who are enrolled in the nutritional programme [28]. The information includes how to (1) conduct a periodic nutritional assessment at a health facility; (2) increase intake and dietary diversification; (3) maintain a good level of hygiene and sanitation; (4) increase clean and safe water intake; (5) maintain a healthy lifestyle; (6) early treatment and diagnosis of illness and symptoms and (7) adherence to advice by a health provider on how to take medications and manage dietary needs. Despite the potential benefits of nutritional counselling to people living with HIV, there is little evidence about the practice and experiences of nutritional counselling in HIV care settings from the perspectives of beneficiaries, service providers and programme managers. A study conducted in Ethiopia examined the availability of nutritional education in a nutritional programme in HIV care but did not examine the experience of various stakeholders of the nutritional counselling [29]. This paper presents the experiences of different stakeholders on nutritional counselling in HIV care settings in Ethiopia, with a view to help inform policy on how to effectively address the challenges of nutritional counselling for people living with HIV.

### The socioecological model

This study draws on the socioecological model (SEM) to assist an understanding of a problem at multiple levels [30]. The SEM is a theory-based framework employed to comprehend the interaction of individual and environmental factors that impact health behaviours and outcomes [31, 32]. The model identifies that there are individual, interindividual, institutional, community and social policy level factors that influence health outcomes and behaviours, and highlights the importance of considering the dynamics and interactions between these levels [33]. The SEM has been applied in studies involving nutritional programmes in HIV care, sexual and reproductive health amongst people living with HIV, adherence to ART and acceptability of infant feeding to prevent HIV [34–37].

This paper sought to examine experiences of the nutritional counselling programme in nutritional programmes in HIV care settings in the Tigray region, from the perspectives of programme users, staff and managers, to identify strengths and challenges of the nutritional counselling that is offered in HIV care.

## Methodology

### Study design

The current study was part of a larger study that examined the determinants of nutritional outcomes and challenges of a nutritional programme in the Tigray region of Ethiopia, which has been described in detail elsewhere [37, 38].

### Sampling and recruitment

This paper presents findings from a qualitative study involving individual interviews with adults living with HIV, caregivers of children living with HIV, health providers and health managers to explore their experiences and perspectives about nutritional counselling in the nutritional programme in HIV care settings.

The study was conducted in three hospitals, namely: Mekelle, Shul and Lemlem Karl hospitals in the Tigray region, northern Ethiopia. These hospitals were selected, within the resource constraints of the study, as representing a key urban hospital and two regional hospitals to reflect different hospital settings. Together, these hospitals constitute to 647 of 6994 (Mekelle, *n* = 419; Shul, *n* = 98 and Lemlem Karl, *n* = 130) of those enrolled in the nutritional programme in the Tigray region who accessed HIV and nutrition-related services.

Study participants consisted of 20 adults and 15 caregivers of children living with HIV enrolled in the nutritional programme—these patients had been enrolled in the programme as a result of anthropmetery measurements. In addition, 11 health providers and two programme managers were interviewed. Details of the study setting are described elsewhere [38].

### Data collection procedure

The first author (FT) conducted face-to-face in-depth interviews with participants. There was no relationship between the interviewer (first author) and the study participants prior to the interview date and participant information sheet and consent form were the only source of information for participants to know about the research interviewer (FT). Semi-structured interview guides were developed (in English) and pre-tested in the field, before their use to collect the current study data (see Supplementary file 1). To induce open discussion on the topics and maintain the privacy of the study participants, interviews were conducted in private rooms inside the relevant health facilities. The first author (FT) who is a native speaker and fluent in English with a good understanding of the study context and culture, conducted all in-depth interviews in Tigrigna local language, audio-recorded them using a high definition audio recorder and documented all the field observations. The duration of the in-depth interview ranged from 30-66 min.

### Data analysis

Interviews were translated and transcribed verbatim from Tigrigna to English by the first author (FT). A translation and transcription accuracy test was done to improve the quality of translation from the local language to English using a person who was fluent in the Tigrigna local language and English, with accuracy rated as high.

Data were analysed using a thematic framework analysis approach [39]. Interview transcripts were read and re-read to gain a holistic understanding of the range of responses and scope of counselling experiences. A list of codes was developed based on four transcripts which served as a framework for analysis and 11 nutritional counselling related working codes were generated. English translated transcripts were imported to NVivo 11 software for analysis. In the second phase of coding, the number of codes that emerged from all transcripts was increased to 14. Finally, these codes were merged, categorised and re-categorised to develop four themes to answer the research question. Coding and data analysis were done inductively by one person, and three transcripts were double coded by co-authors and disagreements were discussed in a team meeting to reach consensus. The socioecological model was used to guide the overall data analysis and the development of themes, presentation and interpretation of findings—reflecting individual, interindividual, institutional, community and social policy factors.

## Results

### Characteristics of study participants

Participants’ mean ages with standard deviation was 37.2 ± 9.7 for adults living with HIV (hereafter ‘adults’), 36 ± 7.3 for caregivers of children living with HIV (hereafter ‘children’) and 35.3 ± 8 for programme staff. All caregivers and 12 (60%) of adult study participants interviewed were female.

The majority of adults (60%) and caregivers (73%) were urban residents. Whilst four (20%) and five (33%) of adults and caregivers respectively attended no school, 10 (50%) and five (33%) adults and caregivers respectively had attended primary school. The average income in adults and caregivers ranged from no reliable income (*N* = 9, 4 caregiver and 5 adults) to 6000 (*N* = 3) (equivalent to 458 Australian Dollars) per month which is much lower than the average annual per capita income in Ethiopia (857 Australian Dollars) for the year 2018. At the time of the interview, six (30%) adults were single and six (30%) married, and nine (60%) of caregivers were married. An equivalent number of males and females were represented when it comes to health care providers and programme managers, and they all had achieved education level at BSc and above (Table 1).

**Table 1:** Sociodemographic characteristics of study participants

Variables		Participants		
		Adults, ***n*** = 20	Caregivers, ***n*** = 15	Programme staff, ***n*** = 13
**Mean age**	Mean age	37.2 ± 9.7	36 ± 7.3	35.3 ± 8
**Hospital**	Mekelle	8	6	5
	Lemlem Karl	6	5	3
	Shul	6	4	3
	TRHB	N/A*	N/A	2
**Gender**	Male	8	0	6
	Female	12	15	7
**Residence**	Urban	12	11	13
	Rural	8	4	0
**Educational level**	No education	4	5	0
	Primary	10	5	0
	Secondary	6	5	0
	BSc and above	0	0	13
**Marital status**	Single (never married)	6	1	2
	Married	6	9	9
	Widowed	5	3	0
	Divorced	3	2	1

### Experience of nutritional counselling in the nutritional programme

Four main themes that emerged from the interview data in relation to experiences of nutritional counselling in the nutritional programme are summarised in Table 2 and discussed in detail below.

**Table 2:** Themes emerged from the qualitative analysis

Theme	Theme description
Frequency and duration of the nutritional counselling	This relates to the frequency of nutritional counselling over the course of participants’ enrolment in the nutritional programme. Duration indicates the length of an individual nutritional counselling session.
Contents of the nutritional counselling	This refers to the actual topics discussed during the nutritional counselling
Acceptability of the nutritional counselling	This theme relates to the acceptability of the nutritional counselling and recommendations by participants
Applicability of the nutritional programme	Refers to the actual implementation of the nutritional recommendations given by the health professional
Challenges/barriers in relation to nutritional counselling	This theme identified the challenges of nutritional counselling in HIV care from the perspectives of study participants including applicability

### Frequency and duration of the nutritional counselling

Comments received about the frequency of nutritional counselling ranged from ‘no counselling at all to more than one counselling. The majority of study participants noted that the nutritional counselling was provided as a one-off session, commonly at the beginning of the nutritional programme.

*…At the beginning when they give you [the nutritional support], they [the health providers] tell you that it is a medication: that is all, nothing else after… (Adult male, #7).*

A small number of adults and caregivers reported receiving no nutritional counselling at all and said that they had been sent home only with the nutritional support:

*Maybe since I am coming from far away, I may not reach the teaching session. Otherwise, I have not yet come across any counselling or teaching session (Adult female, #6).*

Health providers had a different view with regard to the frequency of counselling sessions, reporting that it is given at every visit.

*We did the counselling at each visit. For example, when we have many clients. We send them to the case manager [peer counsellors] for counselling about ART adherence, cleanliness, or personal hygiene and so on (Health provider #4)*

Session duration did not emerge from adult patients and caregivers whilst health providers reported differences in session duration between hospitals. They believed that the duration of the nutritional counselling sessions was shorter than recommended due to case overload.

### Contents of the nutritional counselling as experienced by participants

Even though the nutritional counselling is meant to cover the seven elements mentioned in the introduction [40], participants reported that the nutritional counselling focused on three elements: (a) nutritional support (Plumpynut/Plumpysup); (b) dietary practice and (c) sanitation and hygiene, with a particular emphasis on the nutritional support.

In general, the nutritional counselling mainly focused on the nutritional support (Plumpynut/Plumpysup), including why the nutritional support is given and its benefits as stated by a caregiver below:

*They [health providers] told me that my child’s weight has been decreased and told me to give him the Plumpynut properly. They said, "this [the plumpynut] will improve his weight as well as general health status and told me to follow him properly" (Caregiver #5).*

An adult female also reiterated the focus of the nutritional counselling around the nutritional support, specifically on the benefits of the nutritional support in terms of improving weight and well-being.

*They [health providers] counsel me that the Plumpynut contains important nutrients including Plumpynut and vitamins. They told me that it is very important and beneficial for your weight and overall health (Adult female #1).*

Discouraging patients from sharing and selling the nutritional support was also stated as part of the nutritional counselling regarding the nutritional support.

*The health providers also advised us to take it (the Plumpynut) properly and not to share it with others. Selling, sharing or giving it to other persons is not allowed (Adult male #6)*

The second content of focus of the nutritional counselling identified in HIV care were related to dietary practice and increasing the frequency and diversity of foods to improve nutritional well-being. Even though the lack of access to adequate food was a problem in many participants, counselling to diversify dietary practice with whatever food available at home was another element of the nutritional counselling.

*They counsel us about everything. For instance, they tell us ‘it is not only meat that we [herself and her child] should take, we can also take other foods like grains and vegetables which are equally important (Caregiver #4).*

*We counsel them how their dietary pattern looks like and they are counselled to take balanced foods (Health provider #2).*

The third component covered in the counselling sessions was on mechanisms to maintain hygiene and food safety including handwashing, hygienic handling of the nutritional support and other foods as well as consuming cooked foods:

*They [health providers] tell us to maintain our personal hygiene and environmental sanitation, to wash our hands before use of the nutritional support and to use Woha Agar*^1^* to treat water (Caregiver #2).*

*Hygiene, to drink clean and safe water and to use Woha Agar to treat the water or boil it (water) before use, otherwise I didn’t remember any specific counselling session (Adult male #6).*

*The health providers advised us to eat balanced food, drink clean water, and seek care for any sickness (Adult female, #4).*

In a small number of instances, during the nutritional counselling other topics such as minimising stress to improve overall health, well-being and quality of life and ART adherence were also covered.

*About our dietary practice, they told us to take foods that go with the ART medication such as eggs, meat, and milk. As I told you about that, if you have enough you will eat, if not you will use whatever you have with the ART (Caregiver #16).*

### Acceptability of the nutritional counselling

“Acceptability refers to determining how well an intervention will be received by the target population and the extent to which the new intervention might meet the needs of the target population and organizational setting” [41]. The majority of caregivers and adults felt that the nutritional counselling they received was acceptable and benefited them in terms of providing information about the nutritional support.

*Since I understand the benefits of the Plumpynut, I believe that the counselling service has benefited me to take the Plumpynut accordingly (Adult male #6).*

*… counselling is very important. It helps me to understand its [the nutritional support] benefits to him [the child]) and use it properly, on time and get the necessary next ration (Caregiver #1).*

Most adult and caregiver participants reported improved knowledge about food and nutrition as the benefit of the nutritional counselling, as described by an adult male below:

*If you are taking the ART medication or the Plumpynut accepting it will benefit you, you will get the intended benefits. All the counselling is good for me for my weight and health. I fully practice the counselling provided because I understand that all the benefits of counselling are to improve my weight. When they counsel you, they [the health providers] are giving your life not to get sick and weaker (Adult male, #13).*

Sharing the information received from nutritional counselling with other adults and caregivers was an indication of a high level of acceptability amongst people living with HIV. This was noted by an adult male:

*As a volunteer, I also transfer information to people who are taking Plumpynut not to share it instead to consume it to themselves (Adult male #5).*

*I also teach a mother who have children who are taking the Plumpynut not to share it with others (Caregiver #3).*

### Challenges for the provision of nutritional counselling in HIV care setting

A number of challenges were identified for the provision and implementation of nutritional counselling including a lack of understanding by recipients, training issues and inconsistent delivery and a lack of consideration of the context of people’s lives.

Despite the reported acceptability of the nutritional counselling by most adults and caregivers, some health providers said that nutritional counselling was not well implemented by some patients, particularly by older adults. It was believed that this may be partly due to a limited understanding of the nutritional counselling:

*This is because we may not provide them with adequate counselling as well most HIV patients are uneducated which may result in poor or slow understanding of the counselling service (Health providers #1).*

*We give them similar counselling service to all patients, but some people understand you quickly and others didn’t understand you at all despite the intensity of counselling. Some of those who didn’t accept and apply the counselling are those old and uneducated ones who may not pay it adequate attention (Health provider #2).*

Health providers also noted a range of issues related to the training of staff and training materials and inconsistent delivery of the nutritional counselling. The lack of training of health providers related broadly to the general nutritional programme provided in the HIV care service, with implications also for nutritional counselling:

*Issue related to the health system, one is the professional. In order to give good service; their training should also be good. The health professional should know the contents of the two food supplements. The knowledge of the health provider is low (Health provider #10)*

*There are problems related to the health provider especially if they didn’t take training about food by prescription (Health provider #4).*

Adults further reported that there was no detailed assessment of household socioeconomic status and family situation during the nutritional counselling but underlined that they have been told to eat whatever food available at home. An adult female described the lack of socioeconomic consideration of the nutritional counselling below:

*They [the health providers] didn’t say about this [household socioeconomic consideration]. They didn’t ask about what we have and haven’t. They [health providers] only tell us to diversify our dietary practice. There is nobody who asks about our household situation (Adult female #19).*

A lack of detailed assessment of household socioeconomic status and socioeconomic consideration of the nutritional counselling constrained the acceptability, applicability and adherence of the nutritional counselling in HIV care setting with poor access to adequate food, for some participants, a key barrier to fulfilling the key messages promoted in the nutritional counselling:

*About my dietary practice, I think you have to eat what you get at your home; otherwise, you can’t live according to the counselling given here in this health facility (caregiver #16).*

A small number of caregivers did report the experience of assessment of their household socioeconomic status during the nutritional counselling as stated below:

*It [household socioeconomic status] is considered. They ask me and write it down on my card and they have done everything. They know about my income and my job as well as other things about my family (Caregiver #3).*

These overall challenges associated with nutritional counselling were seen by health providers to have a significant impact on programme outcomes:

*Had it been that they apply all the counselling services given to them like the ART medication, they may get the necessary benefits from the nutritional program: as well we may not have default or loss to follow up from the nutritional program (Health provider #6).*

## Discussion

Nutritional counselling in HIV care settings seeks to improve the effectiveness of nutritional programmes via improving nutritional knowledge and programme utilisation. Research has indicated that locally and culturally sound nutritional counselling is vital to enhance and maintain the nutritional status of people living with HIV [24, 42, 43]. Engaging with participants and empowering individuals with essential nutritional knowledge to understand the importance of nutrition to make informed decisions regarding their nutritional options is fundamental for successful nutritional counselling in HIV care settings [14, 19, 20].

The importance of nutritional counselling is stipulated in the Ethiopian national nutritional programme guidelines [44]. Nevertheless, this study has shown that counselling as one of the key components of nutritional programme had a narrow focus, largely limited to a simple one way provision of information. A similar study from Ethiopia provided limited information on the level of engagement with people living with HIV in the counselling sessions, but what it did find was generally poor engagement of programme beneficiaries [45].

Whilst the technical guidelines for nutritional counselling recommends covering seven elements [40], the current study found that nutritional counselling in the HIV care settings covered three elements–counselling about the nutritional support, dietary diversification and food and personal hygiene. Amongst the three elements, the main focus was on the nutritional support, which led people living with HIV to consider and relate the nutritional programme in HIV care only to the nutritional support. This is not in line with the objectives of the nutritional counselling which is more holistic and comprehensive [14]. This finding is consistent with other studies reporting a focus of nutritional counselling on the nutritional support [46].

Disproportional emphasis on the nutritional support was mainly related to the medicalised orientation of the nutritional programme more generally and the nutritional counselling in particular. Medical orientation refers to a focus of the nutritional programme on treating medical malnutrition [47, 48]. The medicalised orientation of the nutritional counselling may contribute to the lack of a holistic approach—limiting the nutritional counselling to clinical malnutrition, and has been found in other studies [49].

According to the findings of this study, the nutritional counselling on the management of nutritional requirements was well received by patients, providing them with information on the nutritional supplements. Health providers did identify less educated and older people as having potential difficulties understanding the counselling. This finding supports Tobi and Voge assertion that reported a lack of understanding of the nutritional counselling service as a barrier to uptake of the nutrition counselling amongst people living with HIV [50]. On the other hand, other studies have also found that nutritional counselling improved understanding of the nutritional programme and their nutritional management and improved nutritional well-being [14, 24, 25].

There is no evidence on the specific duration of nutritional counselling to bring the required behavioural change amongst people living with HIV. However, low number of nutritional counselling sessions and shorter duration were key challenges to effectiveness of nutrition counselling as demonstrated in this study—with most participants reporting one off counselling session and health providers indicating that session times in some contexts are too short. Other studies have also reported a short duration of nutritional counselling sessions as a key barrier to nutrition counselling being effective. For example, a study by Tafesea and colleagues in Ethiopia indicated that a health provider counselled about 38 clients in a day and the average time spent on counselling per client was 3.26 min, which is very short to cover the contents and deliver key nutrition information [29]. Other studies have reported short duration of nutrition counselling sessions and also that shorter sessions means less focus on providing information on prevention [46, 51, 52].

Health providers who provide nutritional counselling need specialised training and knowledge in relation to culturally sensitive foods, disease progression, ART medication, and complications [50, 53]. However, a lack of continuous standardised training of health providers on nutrition for people living with HIV was identified and this finding is consistent with the findings of studies conducted in Ethiopia [29, 54]. A similar study by Kolasa and Rickett indicated that health providers lacked the necessary knowledge and skill to deliver brief and evidence-based nutritional counselling for people living with HIV [55]. This was also reflected in a lack of consistency in providing the nutritional counselling between health providers in the current study. Whilst the national policy framework in Ethiopia indicates the need for nutritional counselling [40, 44], there were no technical guidelines to guide the nutritional counselling except a simple pamphlet which suggested some seven key nutritional information for people living with HIV (see the “Introduction” section) [40]. In addition, there were no training guidelines for health providers.

A key issue also identified as a challenge for nutrition counselling programmes was a lack of focus on the socio-economic context of people’s lives. This study found that food insecurity and poverty were key issues [38] and these affected their capacity to implement some elements of the counselling. Other studies in Kenya [49], South Africa and Ghana [18, 50] have likewise emphasised the importance of considering the individual’s financial and socioeconomic circumstances when providing nutritional counselling.

Guided by the socioecological model [30], this study indicated that effective nutritional counselling can be influenced by multilevel factors that interact with each other. At an individual level, this includes knowledge, cognition, and acceptability of the nutritional counselling for the individual patient [56, 57]. For instance, acceptability of the nutritional counselling operated as an individual level factor that impacted the effectiveness of the nutritional counselling. Acceptability can be further explained by the interaction of individual-level issues such as lack of understanding and social policy level factors such as food insecurity and poverty [35, 58].

Inter-individual level factors such as lack of socioeconomic consideration of the nutritional counselling was also a community-level factor which highly interacts with social policy level factors such as poverty and food insecurity [59]. The nutritional counselling was implemented in a broader socioeconomic environment where poverty and food insecurity was common [56, 60].

Nutritional counselling can also be an institutional level factor [56], where institutional structure, culture and operational issues were important determinants of the nutritional counselling and outcomes. For instance, the nutritional counselling lacked standardised training for health providers as reflected by inconsistencies in the delivery of the counselling amongst and within the health facilities included in the current study. This could be related to health providers’ skills and knowledge about the nutritional counselling and the lack of goal-oriented and individual problem-based focus of the nutritional counselling. Institutional level factors such as offering counselling session on a one-off basis, short duration of each session, lack of nutritional counselling guidelines, and lack of ongoing training of health providers coupled with policy level factors such as the medicalised orientation of the nutritional counselling negatively impacted the effectiveness of the nutritional counselling.

**Fig. 1:**
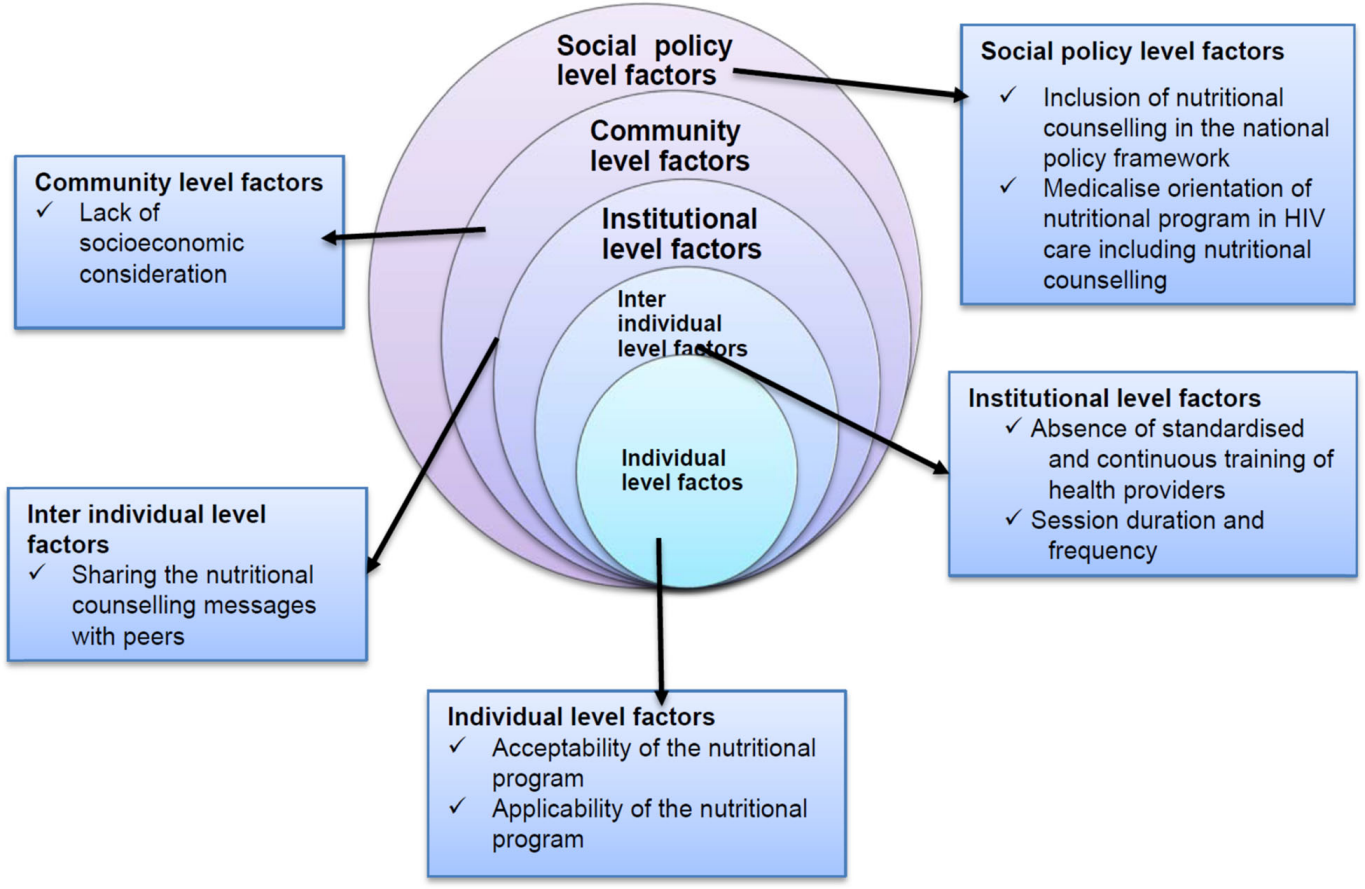
The interrelationships of various factors that impact the nutritional counselling in HIV care settings

There is also an interaction between the institutional level factors which include absence of structured and goal-oriented counselling and policy gap due to the lack of counselling guideline and individual behaviours such as individual needs as stipulated in the socio-ecological model [30] (Fig. 1).

The study was able to canvass the views of the nutritional counselling of people living with HIV and carers of children with HIV, supplemented by interviews with service providers and nutritional programme managers. However, there are a number of potential limitations to the study. There may be issues around maintaining the original meaning of the data during translation and transcription as narrated by the study participants, but a maximum effort was made to maintain accuracy. Although issues of confidentiality were emphasised prior to and during interviews, some participants were concerned about the use of an audio recording device. This may have had an impact on participant responses. The issue of a man interviewing a woman may also constrain some participants to detail some views. However, the interviewer was very mindful in the interviews of being sensitive to this.

## Conclusion and implications

Compared to national guidelines, we found that nutritional counselling in HIV care is characterised by limited scope and is oftentimes provided as one-off session usually at enrolment to the nutritional programme. The other challenges of the nutritional counselling were lack of comprehensiveness, consistency, and regularity, difficulties of some recipients in understanding the recommendations, and a failure to respond to the circumstances of people’s lives.

Comprehensive nutritional counselling that is locally tailored and takes individual patients’ needs and their socio-economic factors into consideration is crucial to improve nutritional health and well-being amongst people living with HIV. The development and improvement of national guidelines on nutritional counselling should include strategies and mechanisms that facilitate their translation into practice. Health providers’ training, and reorientation of the nutritional counselling towards a holistic approach that engage and empower patients is also crucial to enhance the nutritional well-being of people living with HIV.

## Supplementary Information

**Additional file1: Supplementary file 1.** Interview guide.

## Data Availability

The datasets used and/or analysed during the current study are available from the corresponding author on reasonable request.

## Footnotes

1 Woha Agar is a water treatment chemical used in Ethiopia mainly distributed by health providers to people living with HIV.
