# Supplementary material for: Experience of nutritional counselling in a nutritional programme in HIV care in the Tigray region of Ethiopia using the socio-ecological model
Source: J Health Popul Nutr. 2021 Jul 28;40:34. doi: 10.1186/s41043-021-00256-9 (PMC8317349; doi:10.1186/s41043-021-00256-9)
Supplement: Supplementary file 1 — Additional file1: Supplementary file 1. Interview guide. [file 41043_2021_256_MOESM1_ESM.docx]

| **Table 1: Interview guide for adults, caregivers, health providers, and program managers** |
| --- |
| 1. Can you tell me a bit about your HIV condition, family, and the services you get for your HIV?   Probing questions   - How long has it been since your diagnosis? - Are there other family members living with you who have HIV? - What services/treatments are you getting for your HIV? (Probe: ART, nutritional assessment, nutritional counselling, and provision of therapeutic/supplementary food?) |
| 2. What are the best services you received from this clinic in relation to weight and nutrition problems so far? Why? |
| 3. Now, I will ask you about the nutritional counselling you get in the nutritional program?  Probing questions   - How helpful is the nutritional counselling to maintain your weight? - How the nutritional counselling given here does benefit you in relation to the supplementary/therapeutic food? - Do you think the health provider considers your household or life conditions when providing counselling? - What are the components of the nutritional counselling given to you here? - Can you describe to me a typical counselling session (probe)? - How and when did the nutritional counselling given to you in this HIV clinic? - Have the health providers explained to you how and when to use the supplementary/therapeutic food? Are you given the chance to ask questions or the challenges you have? |
| 4. What are the challenges you face to apply the nutritional counselling? |
| 5. Have you been in the program before and what is your previous history/experience? |
| 6. What are the challenges/ difficulties you faced for being put at the program? How did you overcome them or how would you support to overcome them?   - How do you think the program can be improved? |

**Interview guide**
